# Design and testing of a mobile health application rating tool

**DOI:** 10.1038/s41746-020-0268-9

**Published:** 2020-05-21

**Authors:** David M. Levine, Zoe Co, Lisa P. Newmark, Alissa R. Groisser, A. Jay Holmgren, Jennifer S. Haas, David W. Bates

**Affiliations:** 1 Division of General Internal Medicine and Primary Care, Brigham and Women’s Hospital, Boston, MA USA; 2 Harvard Medical School, Boston, MA USA; 3 Department of Clinical Quality and Analysis, Partners Healthcare System, Somerville, MA USA; 4 Harvard Business School, Boston, MA USA; 5 Division of General Internal Medicine, Massachusetts General Hospital, Boston, MA USA

**Keywords:** Health policy, Health services, Diagnosis, Therapeutics

## Abstract

Mobile health applications (“apps”) have rapidly proliferated, yet their ability to improve outcomes for patients remains unclear. A validated tool that addresses apps’ potentially important dimensions has not been available to patients and clinicians. The objective of this study was to develop and preliminarily assess a usable, valid, and open-source rating tool to objectively measure the risks and benefits of health apps. We accomplished this by using a Delphi process, where we constructed an app rating tool called THESIS that could promote informed app selection. We used a systematic process to select chronic disease apps with ≥4 stars and <4-stars and then rated them with THESIS to examine the tool’s interrater reliability and internal consistency. We rated 211 apps, finding they performed fair overall (3.02 out of 5 [95% CI, 2.96–3.09]), but especially poorly for privacy/security (2.21 out of 5 [95% CI, 2.11–2.32]), interoperability (1.75 [95% CI, 1.59–1.91]), and availability in multiple languages (1.43 out of 5 [95% CI, 1.30–1.56]). Ratings using THESIS had fair interrater reliability (*κ* = 0.3–0.6) and excellent scale reliability (*ɑ* = 0.85). Correlation with traditional star ratings was low (*r* = 0.24), suggesting THESIS captures issues beyond general user acceptance. Preliminary testing of THESIS suggests apps that serve patients with chronic disease could perform much better, particularly in privacy/security and interoperability. THESIS warrants further testing and may guide software and policymakers to further improve app performance, so apps can more consistently improve patient outcomes.

## Introduction

Mobile health applications (“apps”) have proliferated more rapidly than almost any other innovation in health care: over 300,000 health apps are available today, representing a doubling since 2015. An estimated 40% of all apps (up from 27% in 2015) are related to health^1^. This has been made possible by the rapid adoption of app-enabled mobile telephones, from 35% of Americans in 2011 to 77% in 2018^2^. In 2015, national surveys suggested that more than half of mobile device users have downloaded a health app, although this does not connote use^3,4^.

Despite this proliferation, few health apps have been shown to achieve what is arguably their most important goal: to improve patient outcomes^5–7^. Many apps appear to be focused on relatively healthy patients, with many fewer being focused on high-cost, high-need patients, or patients with chronic diseases^8^. Instead, most apps are used for short periods of time and then dropped^9^. This is problematic especially for patients with chronic diseases who may benefit from a longer-term experience. We know that comprehensive longitudinal care affords patients better outcomes^10^, but a longitudinal relationship with an app is not the norm. When it does exist, for example, significant reductions in hemoglobin A1c in type 1 diabetes have occurred^11^. While short-term use of an app may be effective, such as for patients undergoing colonoscopy who have better colonic prep with the guidance of an app^12^, we focused on chronic disease given its large burden on society and opportunity for improvement^13–15^.

Despite their potential benefits, apps also carry risks. Some apps have even caused harm, whether by misdiagnosis of skin cancer or incorrect reporting of blood pressure^16–20^. Even those that are not directly harmful may have lax standards regarding security, interoperability, and health content that could cause harm not yet realized by the user. Many app users are left with little more than an app’s star rating to decide whether an app may be right for them. Few published studies evaluate unintended adverse events caused by app use^8^. A rating tool that seeks to identify apps that may cause harm could improve the safety of apps and reduce future adverse events.

We hypothesized that multiple specific dimensions can be identified around which apps can be characterized to describe their quality, safety, and potential value to patients and clinicians. If this is the case, a standardized, usable rating tool could help enable consumers and clinicians to make informed decisions regarding app use. It could also guide app developers, regulators, and policymakers. A number of efforts have been made to do this for apps, but they have been stymied by poor usability and an incomplete evidence-base, among other issues^21^.

With the right combination of usability, validity, security, and privacy, we believe health apps can be beneficial, and even potentially transformational for health and health care. However, tools to address these and other dimensions are not widely used, and routine ratings addressing these dimensions are not available resulting in a lack of information to make informed decisions on which apps to use or recommend^22^. As a step toward addressing this issue, we developed and preliminarily assessed a rating tool to objectively assess the risks and benefits of health apps. We present the tool development process, tool characteristics, and preliminary ratings produced using the tool with several hundred of today’s health apps, some highly rated by conventional star rating systems, as well as lower-rated apps, to see if we could distinguish differences.

## Results

### Expert panel

The expert panel included nine individuals; three participated only in pre-panel rating, while six participated in both pre-panel rating and the panel on September 8, 2017. Panel members included a patient representative and experts in health communication, computer science, and health care technology (Supplementary Table 1 for list of participants).

Panel discussion after each private ranking resulted in both deletions and additions to the criteria (Supplementary Method 1 for iterations of the tool). Panelists felt that the relative importance of one domain compared to others was difficult to assess despite being tasked with ranking each. New criteria added by the panel included bandwidth and memory requirements, as these may impact a user’s ability to download and use apps. The panel removed the requirement of a privacy statement, noting a statement was much less important than the proper safeguard built into the app. This process finalized into a rating tool with 27 items and six overarching domains (Table 1 and Supplementary Table 2).

**Table 1:** Mobile health app rating domains and criteria.

Domain	Criteria
Transparency	Cost of app Consent Accuracy of app store description
Health content	Appropriate measurement Appropriate interpretation of data Quality of information Potential for harm Literacy level Presentation of information
Technical content	Software performance/stability Interoperability Bandwidth Application size
Security/Privacy	Protection against theft and viruses Authentication Data sharing Maintenance Signaling of breaches Anonymization
Usability	Installation and setup Functionality Aesthetics Customization/tailoring Ease of use for users with low literacy and numeracy Availability in multiple languages
Subjective	Recommend app Overall star rating

Refer to Supplementary Table 3 for detailed descriptions of each individual item.

The final overarching domains were *t*ransparency, *h*ealth content, *e*xcellent technical content, *s*ecurity/privacy, *i*ssues of usability, and *s*ubjective ratings, forming the acronym THESIS.

### Apps identified

**Fig. 1:**
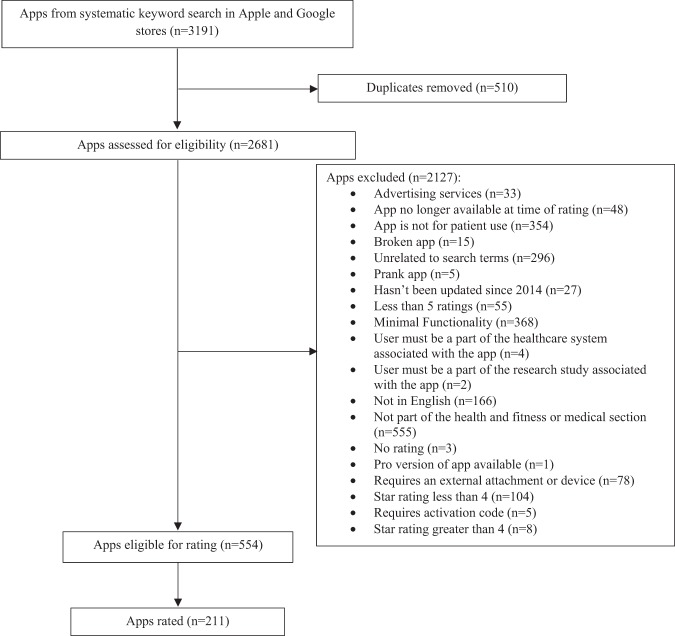
App selection (all categories combined). We selected the first four apps in each disease category. Not all apps were rated due to resource constraints. Please refer to Supplementary Figs. 1–3 for individual category selection details.

Of the 3191 initially identified apps, we evaluated 211 (Fig. 1). Of the 137 category 1 apps evaluated in our prior study, only 37 were still available or met criteria (Supplementary Fig. 1)^23^. For category 2, we evaluated 1350 apps and rated 88 apps (Supplementary Fig. 2). For category 3, we evaluated 1704 apps and rated 86 apps (Supplementary Fig. 3).

### App ratings

**Fig. 2:**
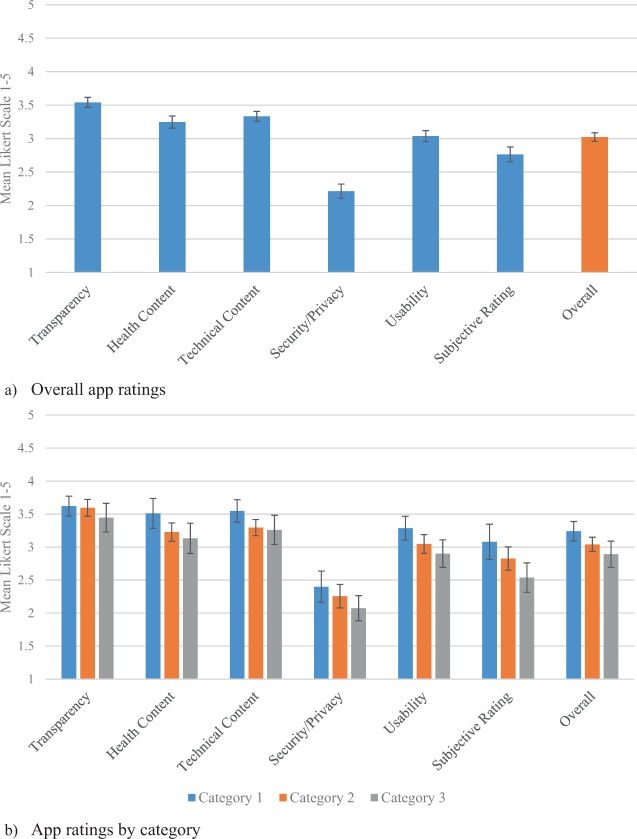
App ratings. **a** Overall app ratings. **b** App ratings by category. The error bars represent 95% confidence intervals. See Supplementary Table 4 for detailed ratings.

The mean overall app rating was 3.02 out of 5 (95% CI, 2.96–3.09) (Fig. 2a). Significant differences existed among domains (*p* < 0.01), most notably with security/privacy receiving the lowest rating by far: 2.21 out of 5 (95% CI, 2.11–2.32). In contrast, the most highly rated domain was transparency: 3.54 out of 5 (95% CI, 3.47–3.62). Other individual items (Supplementary Table 3) with particularly low ratings included consent (1.86 out of 5 [95% CI, 1.73–1.98]), interoperability (1.75 out of 5 [95% CI, 1.59–1.91]), and provision in multiple languages (1.43 out of 5 [95% CI, 1.30–1.56]).

By category, similar between-group differences were evident as in the overall analysis (*p* < 0.01) (Fig. 2b). Within-group differences consistently trended with category 1 apps being the highest rated, followed by category 2, and lastly category 3 but were not statistically significant. For example, for security/privacy, category 1, 2, and 3 had ratings of 2.40 (95% CI, 2.16–2.64), 2.26 (95% CI, 2.08–2.43), and 2.07 (95% CI, 1.89–2.26), respectively.

By disease condition, similar between-group differences were evident as in the overall analysis (*p* < 0.01). The two lowest-rated conditions were human immunodeficiency virus (HIV) (mean 2.43; 95% CI, 1.67–3.9) and schizophrenia (mean 2.54; 95% CI, 2.31–2.76). The two highest-rated conditions were chronic obstructive pulmonary disease/asthma (mean 3.35; 95% CI, 0.31–6.39) and obesity (mean 3.36; 95% CI, 2.69–4.03).

Each rating required a mean of 13.9 min (95% CI, 13.24–14.48 min) to complete.

### Tool and star rating correlation

Of the 211 apps evaluated, 154 had conventional star ratings. Our overall ratings had a low correlation with app store ratings (Spearman’s *r* = 0.24; *p* < 0.01). Apps rated 4 stars or more had a mean overall rating of 3.04 (95% CI, 2.93–3.15) compared to 2.89 (95% CI, 2.80–2.98) for apps rated less than 4 stars (*p* = 0.10).

### Interrater reliability and internal consistency

The Cohen’s kappa varied by domain: transparency (0.49), health content (0.31), technical content (0.60), security/privacy (0.46), usability (0.50), and subjective rating (0.33). Cronbach’s alpha was 0.85, demonstrating an adequate level of scale reliability and internal consistency.

## Discussion

We describe the construction process, characteristics, and preliminary test characteristics of a novel mobile health app rating tool. When we used the tool to assess an array of chronic disease apps, we found that their performance was mediocre overall, and they received strikingly low marks on security/privacy, consent, and interoperability. These areas represent opportunities for improvement for app developers. We envision that in the future THESIS might serve as a robust method for app evaluation that might be scored by both health care professionals and lay users. Its output would be useful for both as well.

Our work corroborates and builds on prior app rating tools^24–39^. While some tools focus mainly on usability and others on security and privacy, we attempted to holistically evaluate the clinically meaningful aspects of an app. Our work also supports prior findings that apps are not particularly serving high cost high need patients, exemplified by HIV and schizophrenia being two of the lowest performing disease categories. Our expert panel identified some novel rating items such as bandwidth requirements.

Apps were often transparent about their intended use and cost, but not about consent for use of data. Apps would sometimes present high-quality information or take appropriate measurements but would less often appropriately interpret data. Their technical content was excellent regarding software stability, but some apps required significant bandwidth and device storage, and very few apps were interoperable with other applications. Apps struggled with delivering strong authentication requirements and signaling of breaches, with poor protection against viruses or clear maintenance schedules. Apps installed well but were not easily used by those with lower health literacy and were rarely available in multiple languages. Fewer apps were available for conditions more often stigmatized. Overall, our raters gave apps a subjective star rating of 3 out of 5, even though our sample included many apps which received high ratings in the app store.

Notably, an enormous turnover of apps occurred between our prior work 2 years ago and 2018 (of the 137 category 1 apps evaluated in our prior study, only 37 were still available or met criteria). Even during the rating process (5 months), some apps became unavailable in the app store. This calls into question the sustainability of apps as they are being developed today. Another concern is whether they could cause harm because of their availability which is typically short-term, akin to prescribing medicine or using a medical device only available for a few months yet is expected to be available for years. This does depend on the intended use of an app—if the app is just intended for use around a procedure, or to provide tips during an exacerbation of a condition like an acute episode of depression, this transience could be acceptable. But if the app is intended for long-term management of a chronic condition, it would be less so.

Traditional app store star ratings poorly correlated with our tool’s rating. This could be because a single item does not capture all the dimensions involved in a tool such as this, or because some users do not value issues such as security/privacy or interoperability, or even because some of the ratings are not supplied by actual users^40,41^. While star ratings work quite well for issues like restaurants or hotels, and they have been found to correlate well with traditional quality rankings in healthcare, they may not be sufficient for apps^42^.

Our tool may be useful to both policymakers and software developers. On the policy front, the Food and Drug Administration has created a “precertification” program for mobile apps, and it is intended to update the regulatory framework enacted by Congress some years ago targeting hardware^43,44^. The prior framework has not worked well with software which changes frequently. The “precert” program is intended to consider these rapid changes. However, its main target so far appears to be the robustness of the software which is being developed. It has not included to date an approach to enable third-party assessments of apps, which seems to be a key need in the marketplace. Moreover, there is no focus so far to make sure the apps make a difference—that is to say, are usable by patients and change health outcomes.

The tool may also be useful for app developers as it can provide a checklist of issues to be addressed. Clearly, privacy, security, and interoperability are major issues, and more data about which apps affect clinical outcomes are needed.

More broadly, policies are needed to incentivize developers to build apps that would score highly on the tool’s various items. For example, requiring that apps have industry-standard security protection and turnkey interoperability could swiftly change today’s offerings.

The tool’s poor alignment with star ratings suggests that more than a star rating should be used to drive users to apps. We envision a curated and/or crowd-sourced version of this tool to assess apps and more-appropriately drive downloads, which would ideally be held in a public-private partnership.

Providers can also work with patients to help them find apps that may benefit them. We believe that one role of specialty societies may be to identify apps that they consider to be high-quality in their domain. Many societies are taking this on. Similarly, integrated delivery systems may wish to identify apps that they consider beneficial and that exchange data readily with their clinical systems.

This analysis has limitations. First, our rating tool may not address every important aspect of app evaluation. However, given our desire to create a tool that did not require hours to complete (we found about 12 min required for completion) and the limited evidence base, our use of an expert panel and Delphi process appeared to be the best option. Second, while we involved a very diverse set of stakeholders in designing THESIS, we were only able to enlist three preclinical raters to preliminarily examine its validity due to resource constraints. We were therefore unable to capture the underlying sociodemographic variations of the millions of app users who gave star ratings. We plan to improve our validation of THESIS with a much larger cohort of raters from varied backgrounds, including clinicians, technology experts, and patients. This will likely address the only-moderate inter-rater reliability we found. Third, inherent in developing a rating tool in a fast-paced and highly variable app landscape is that a relevant factor today may in the future become obsolete or new factors may later emerge as relevant. We note that our data are, by the time of this publication, already potentially outdated. However, we anticipate that the rating tool will serve as a framework to build upon as innovation and adoption increases. Additional dimensions could readily be added if necessary. Fourth, given the thousands of health apps, we rated a relatively small number of apps. We may have missed apps due to our search criteria, although we rated more apps than most prior studies in this field and used prespecified systematic search criteria. Fifth, although we assessed usability, we did not address potential gaps in the full complement of digital skills needed to navigate apps^45^.

A multi-stakeholder group identified methods for rating health apps, forming the THESIS tool. Preliminary testing of THESIS suggests apps perform poorly especially for privacy/security and interoperability, and few appear to be intended for patients with chronic conditions. THESIS warrants further testing and may guide software and policymakers to further improve app performance.

## Methods

### Tool development

**Fig. 3:**
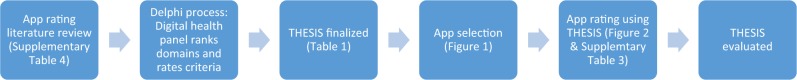
Path to building and evaluating THESIS. The methods taken in the development and evaluation of THESIS. Apps from systematic keyword search in Apple and Google stores (*n* = 3191).

We first performed a review of prior guidance documents and tools used to rate apps to identify previously considered overarching domains and individual items (Fig. 3)^24–39^. Over a dozen different guidance documents and tools are available, each with their own benefits, particular niche, and room for improvement (Supplementary Table 4). For example, the Mobile App Rating Scale (MARS) is a well-studied tool to rate apps covering a wide range of domains related to quality. Areas not addressed by MARS concern privacy, security, interoperability, and access. Our hope was to improve on prior documents and tools such that in late 2017, we invited a panel of digital health experts from industry, academia, and patient groups from across the US to participate in a modified Delphi process to review and prioritize the metrics for a rating tool^46^. Prior to convening the expert panel in-person, we sent each participant the overarching domains and individual items inside each domain identified from our literature review and own team’s expertise (“pre-panel”). We asked each participant to anonymously rank the domains by importance (1 = most important; 6 = least important) and to rate the criteria for each domain (Likert scale 1 [not important] to 5 [absolutely essential]).

During the in-person meeting, we presented these data for group discussion. We then asked participants to again privately rank domains and rate individual items. We presented these updated data for a final round of discussion and a final private ranking and rating. We used these data to develop the final rating tool (Supplementary Method 1 for iterations). The panel anticipated that users of the rating tool would require a college-level education or a highly tech-trained background to perform the ratings.

### App selection

Our goal was to include a diverse set of apps that were of higher and lower star rating, recognizing that app store ratings might not reflect actual quality. To ensure a diverse set of apps, we had three prespecified app rating categories, all of which focus on apps for chronic disease^43^. We included highly rated apps in our prior study (category 1), apps rated 4 or more stars (category 2), and apps rated less than 4 stars (category 3). For all three categories, we excluded apps that were not in English, were removed from the app store during the study period, were primarily selling a product other than the app, had minimal functionality, lacked updates since 2014, required an external device or attachment (due to funding constraints), required association with a health system account, or were not in the health and fitness or medical sections (Fig. 1). Apps available for both Apple and Android were reviewed only on the platform that they were first released. Apps in categories 2 and 3 all had large numbers of star ratings to avoid variations on the average app rating.

Category 1 were apps chosen from prior work that through different methodology were noted to perform well for chronic disease and high need high cost patients^8,23^.

Category 2 were apps for chronic disease rated 4 stars or more in their respective app store. We searched both app stores with the name of the chronic disease (for example, “hypertension”) and selected the four most highly rated apps for each chronic disease. If an initial search did not yield sufficient apps, we instead searched a prespecified reflex term (for example, instead of “hypertension” we searched “blood pressure”). If an initial search yielded apps that were not pertinent, we addended the search term with prespecified modifiers (for example, “blood pressure manager”). We searched for hypertension, heart failure, coronary artery disease, cardiac arrhythmia, hyperlipidemia, stroke, arthritis, asthma, cancer, chronic kidney disease, chronic obstructive pulmonary disease, dementia, cognitive impairment, depression, diabetes, hepatitis, HIV, osteoporosis, schizophrenia, bipolar disorder, substance use disorder, and pain. Not all eligible apps were rated due to resource constraints. We attempted to rate the four most highly rated apps in each disease group, but if there were insufficient apps, we rated additional apps in disease groups already with 4 ratings (Supplementary Method 2 for detailed search criteria).

Category 3 were apps for chronic disease rated less than 4 stars in their respective app store. We similarly searched both app stores with the name of the chronic disease and prespecified reflex terms, as in category 2. We selected the first 50 non-5-star-rated apps and randomly selected 4. If there were insufficient apps in each condition, we rated as many as were available (Supplementary Method 2 for detailed search criteria).

### App rating

We had three raters (one medical student, one pre-medical student, and one business graduate student) use the tool. Two raters rated each app in each category. Each domain had criteria (Table 1 and Supplementary Table 2). Each criterion was rated on a 5-point Likert scale by the two raters. To combine the two rater’s individual ratings, we calculated the mean for each criterion. We calculated the overarching domain score by computing the mean of the combined criteria ratings. We then calculated the app’s overall rating by computing the mean of the overarching domain scores.

We created a norming tool to help ensure raters would rate apps in a similar fashion (Supplementary Method 3). The norming tool detailed how to properly rate an app and gave the rater reasoning for the correct score, and why the score was not one less or one more in each category of the tool. We tracked how many minutes each rating required. Apps were evaluated between December 2017 and May 2018.

### Data analysis

All methods were approved by the Partners HealthCare Institutional Review Board and informed consent was obtained from participants.

We used descriptive statistics to evaluate the app ratings. We evaluated ratings overall, by overarching domain, by category, and by chronic disease.

We used ANOVA to determine if there were significant differences among groups. We examined how our overall ratings aligned with star ratings in the app store with a spearman correlation. We examined interrater reliability with a weighted Cohen’s kappa and internal consistency with Cronbach’s alpha, performed at the domain level. We considered *p* < 0.05 to be significant. We used SAS v9.4 for all analyses.

### Reporting Summary

Further information on research design is available in the Nature Research Reporting Summary linked to this article.

## Supplementary information

Supplementary Information

Reporting Summary

## Data Availability

The datasets generated during and/or analyzed during the current study are available from the corresponding author on reasonable request.

## Code availability

We used SAS 9.4 for simple statistics that are described in the Methods section. This code would be made available upon reasonable request.
