# Supplementary material for: Design and testing of a mobile health application rating tool
Source: NPJ Digit Med. 2020 May 21;3:74. doi: 10.1038/s41746-020-0268-9 (PMC7242452; doi:10.1038/s41746-020-0268-9)
Supplement: Supplementary file 1 — Supplementary Information [file 41746_2020_268_MOESM1_ESM.pdf]

SUPPLEMENTAL INFORMATION

Supplementary Table 1: Expert panel participants
Supplementary Table 2: Detailed app rating criteria
Supplementary Table 3: Detailed ratings
Supplementary Table 4: App rating tools and guidance documents
Supplementary Figure 1: Category 1 (prior study) app selection
Supplementary Figure 2: Category 2 (4 stars or more) app selection
Supplementary Figure 3: Category 3 (less than 4-star) app selection
Supplementary Method 1: Tool iterations during Delphi process
Supplementary Method 2: Detailed search criteria
Supplementary Method 3: Norming Tool

Supplementary Table 1. Expert panel participants

Name	Participation	Affiliation
Martie Carnie	Pre-group and group	Co-Chair, Patient Family Centered Care Steering Committee / Senior Patient Advisor<br>Brigham and Women's Hospital
Deborah Estrin, PhD	Pre-group and group	Professor of Computer Science and Associate Dean, Cornell Tech
Chandra Y. Osborn, PhD, MPH	Pre-group and group	Vice President of Health & Behavioral Informatics, One Drop, Informed Data Systems Inc.
Nina Nashif, MS	Pre-group and group	Managing Partner, Generation Health
William D. Terry, MD	Pre-group and group	Director, Center for Interdisciplinary Cardiovascular Sciences, Brigham and Women’s Hospital
K. Vish Viswanath, PhD	Pre-group and group	Faculty, Harvard University; Faculty Director, Health Communication Center, Dana-Farber/Harvard Cancer Center
Aaron Seib	Pre-group	CEO, National Association for Trusted Exchange
Barbara J Grosz, PhD, MA	Pre-group	Higgins Professor of Natural Sciences, School of Engineering and Applied Science, Harvard University
Malay Gandhi	Pre-group	Senior Vice President of Business Operations, Evidation Health; Former CEO, Rock Health
Eric C. Schneider, MD, MPH	Representative of the Commonwealth Fund	Senior Vice President for policy and research, The Commonwealth Fund

Supplementary Table 2. Detailed app rating criteria

- **Transparency**
    - Cost of app (purchase price, subscriptions, in-app purchases)
        - Are the prices, subscriptions, and in-app purchases accurately conveyed?
    - Consent
        - What is the quality of the consent process, if any?
    - Accuracy of app store description
        - How accurate is the app store description of the app's purpose?
- **Health content**
    - Appropriate measurement
        - Does the app appropriately measure what it claims to measure?
    - Appropriate interpretation of data
        - Does the app appropriately interpret what it claims to interpret?
    - Quality of information
        - How optimal is the quality of information?
    - Potential for harm
        - Is the potential for harm minimized?
    - Literacy level
        - How appropriate is the literacy level for the app's intended audience?
    - Presentation of information
        - Is information presented in an optimal manner? For example, is scaffolding used?
- **Technical content**
    - Software performance/stability
        - Does the app run well with zero interface crashes or bugs?
    - Interoperability
        - Is the app able to exchange information with EHRs and other apps?
    - Bandwidth
        - Does the app require significant bandwidth to run?
        - 5: App does not require the use of cellular data; very few graphics used in the app
        - 4: Main function of the app doesn't require the internet or cellular data, however there is a decent number of images or animations used in the app
        - 3: App uses large images and animations, and only a few of its functions require the use of internet or cellular data
        - 2: Many of the app's function use the internet or need cellular service, however the user can use the app offline

        - 1: The main functions of the app require significant use of cellular data, access to the internet and/or location services; app includes a large amount of images, animations, and/or videos. User cannot use app without internet (no offline version).
    - Application size
        - Does the app require significant storage capacity?
        - 5: <10 MB, 4: between 10 to 20 MB, 3: between 20-30 MB, 2: between 30-40 MB, 1: >40 MB
- **Security/privacy**
    - Protection against theft, viruses, etc
        - Does the app follow best practices in security with optimal anti-virus and safeguards against breaches?
    - Authentication
        - Is the authentication procedure optimal?
    - Data sharing
        - When sharing information, does the app use best practices?
    - Maintenance
        - Does the app have regular cycles to update and patch its security?
        - 5 – last update occurred during the month of rating or during the month before; also, if the update schedule is very consistent
        - 4 – last update occurred 2 months before the time of rating; update schedule is generally consistent
        - 3 – last update occurred between 3-5 months before the time of rating; update schedule is a little inconsistent
        - 2 – last update occurred between 6 months to a year before time of rating; update schedule is completely inconsistent
        - 1 – last update was occurred more than 1 year ago
    - Signaling of breaches
        - If a breach occurs, does the app have a method to notify its users?
    - Anonymisation
        - Does the app appropriately anonymize individuals?
- **Usability**
    - Installation and setup
        - How would you rate are installation and setup?
    - Functionality: ease of use, navigation, gestural design, help/instructions
        - Quality of ease of use, navigation, gestural design, help/instructions?
    - Aesthetics: layout, graphics, visual appeal, image readability
        - Quality of layout, graphics, visual appeal, and image readability?
    - Customization/tailoring
        - Ability to customize and tailor to the specific user's needs?
    - Ease of use for users with low literacy and numeracy

        - Is the app usable by users with low literacy and numeracy?
        - 5: The medical information provided by the app does not use a significant amount of medical terms; complex conditions are explained using laymen's terms. The medical information is also supplemented with simple images or short animations. Lastly, if the app allows users to input their symptoms, they can use laymen's terms, instead using medical terms, and the app will output the possible conditions the patient may have.
        - 4: Overall, the medical information in the app is sufficiently explained using laymen's terms, and any graphics used to supplement this information is simple and clear. There are very few instances where medical terms are not explained.
        - 3: Some of the medical information in the app uses medical terms that are not sufficiently explained or supplemented by diagrams or images. Not all the information is complex, patient or their caregiver can still navigate through the app.
        - 2: Very few laymen's terms are used to describe medical conditions. Medical terminology is not explained. Any images or graphics meant to help explain medical conditions require some medical familiarity.
        - 1: The language used in the app is complex, and very difficult to understand if the user does not have any prior medical knowledge. The description of medical conditions does not use any laymen's terms. No resources such as images or graphics to help explain medical conditions.
    - Available in multiple languages
        - Is the app available in multiple languages?
        - 5: >16, 4: 12-16, 3: 8-12, 2: 4-8, 1: <4
- **Subjective rating**
    - Recommend app
        - Would you recommend this app?
    - Overall star rating

What is your overall rating of this app?

Supplementary Table 3. Detailed ratings

Domain	Criteria	Mean (95% CI)
**Transparency**		
	Cost of app	4.51 (4.42,4.59)
	Consent	1.86 (1.74,1.98)
	Accuracy of app store description	4.26 (4.16,4.36)
**Health content**		
	Appropriate measurement	3.37 (3.26,3.49)
	Appropriate interpretation of data	2.90 (2.77,3.02)
	Quality of information	2.85 (2.73,2.97)
	Potential for harm	3.36 (3.24,3.48)
	Literacy level	3.85 (3.74,3.95)
	Presentation of information	3.15 (3.03,3.27)
**Technical content**		
	Software performance/stability	4.41 (4.32,4.50)
	Interoperability	1.75 (1.63,1.88)
	Bandwidth	3.61 (3.50,3.71)
	Application size	3.56 (3.42,3.71)
**Security/Privacy**		
	Protection against theft and viruses	2.47 (2.31,2.62)
	Authentication	1.83 (1.71,1.96)
	Data sharing	2.78 (2.63,2.94)
	Maintenance	2.43 (2.30,2.57)
	Signaling of breaches	1.17 (1.10,1.24)
	Anonymization	2.60 (2.46,2.74)
**Usability**		
	Installation and setup	4.44 (4.36,4.52)
	Functionality	3.53 (3.42,3.63)
	Aesthetics	2.99 (2.87,3.11)
	Customization/tailoring	2.71 (2.59,2.84)
	Ease of use for users with low literacy and numeracy	3.13 (3.04,3.22)
	Availability in multiple languages	1.43 (1.33,1.53)
**Subjective**		
	Recommend app	2.55 (2.44,2.66)
	Overall star rating	2.98 (2.89,3.06)

Supplementary Table 4: App rating tools and guidance documents

Tool/Organization	Description	Source/Citation	Strengths	Room for Improvement
MARS	The Mobile App Rating Scale (MARS), is a rating scale (ICC=0.79 and internal consistency: alpha=0.90) used to assess the quality of mobile health apps. The scale contains four overarching domains: engagement, functionality, aesthetics, and information quality, and a section focused on the subjective quality of the app. The mean score from MARS describes the quality of the app, while the individual domain scores pinpoint strong and weak areas.	Stoyanov SR, Hides L, Kavanagh DJ, Zelenko O, Tjondronegoro D, Mani M. Mobile App Rating Scale: A New Tool for Assessing the Quality of Health Mobile Apps. *JMIR mHealth uHealth*. 2015;3(1):e27. doi:10.2196/mhealth.3422.	MARS covers a wide range of domains related to the quality of apps. One of the sections in the tool asks about the potential impact the app could have in changing behavior; a section not included in most rating scales.	MARS didn't cover areas concerning secutiy/privacy, interoperability, or the access of these apps to high-cost high-need populations.
KNMG	The Medical App Checker is a toolkit created by the Royal Dutch Medical Association (KNMG), for doctors to evaluate medical apps they intend to use for themselves, or to recommend to patients and their caregivers. The toolkit is specific for apps that are considered medical devices, apps that track and monitor patient symptoms, and communication apps that enable users to contact their provider. The App Checker is made up of three sets of questions, belonging to areas focused on searching for reliable apps, determining the reliability and quality of the app, and measures taken around the protection and security of patient information. Answering these questions will help doctors determine the quality of the app.	*Medical App Checker: A Guide to Assessing Mobile Medical Apps.*; 2016. https://www.knmg.nl/actualiteit-opinie/nieuws/nieuwsbericht/medical-app-checker-a-guide-to-assessing-mobile-medical-apps.htm. Accessed July 1, 2018.	This toolkit is specific for apps that convert the mobile device to a medical instrument. The questions are very in depth. This toolkit has very detailed reccomendations. For example, for each of the sets of questions, if a user answers "no" for two or three of the questions, it is advised not to use the app.	While this toolkit covers a lot of areas concerning the use and safety of an app, it does not cover areas concerning the cost of apps, and availibility of apps to high-cost high-need populaitons. In addition, the toolkit has reccomendations for when users answer "no" to a certain number of questioons, but do not provide reccomendations for when users answer "Don't know". It requires a large

				time investment to complete.
HAS Santé	The Haute Autorité de Santé consists of an expert committee, who performed a thorough literature review on health apps and smart devices. As a result, they produced a set of practice guidelines, meant to encourage the use of health apps, and provide increased confidence in their use.	*Assessment and Improvement of Practice Good Practice Guidelines on Health Apps and Smart Devices (Mobile Health or MHealth)*. Saint-Denis La Plaine Cedex; 2016. https://www.has-sante.fr/portail/upload/docs/application/pdf/2017-03/dir1/good_practice_guidelines_on_health_apps_and_smart_devices_mobile_health_or_mhealth.pdf. Accessed July 1, 2018.	A great resource for understanding areas that are critical for the safe and effective use of mobile health apps. Plenty of examples given that help further the goals of each section.	Although this report is a great resource, it is not a rating system. The information in this report can be used as guidance.
Medappcare	Medappcare is a research council that focuses on the confidentiality and up to date regulation around mobile health apps. Developers can submit their apps to them, and the app will go through several evaluations (i.e. “medical evaluation” and “technical evaluation”). If the app passes all of these evaluations, Medappcare will add them to their database, where one of their prescribers can recommend the app to users.	https://www.medappcare.com/en/methodologie-devaluation/	A major strength is the “Continuous evaluation by users”, which can provide valuable feedback to the app developers. The evaluators are all healthcare or industry professionals.	The process for getting an app evaluated by this company seems simple. However, more information and resources on what they think is important in “Medical Evaluation” and “Technical Evaluation” are needed to receive a precise understanding of

				the evaluation process.
Calidadapp	Calidadapp is an expert committee that made several recommendations about the design, use, and evaluation of mobile health apps. Their audience involved several groups varying from health professionals to app developers. Design, quality and safety of information, provision of services, and confidentiality and privacy were the areas their recommendations focused on.	http://www.calidadappsalud.com/en/listado-completo-recomendaciones-app-salud/	A great resource to use for developing a mobile app rating tool. The recommendations go into great detail, for example in "Confidentiality and Privacy" section, there are sub sections focused on privacy and data protection, and logical security. Each recommendation is also connected to an external link, where there is more information.	Note, this is more of a resource, and not a mobile app rating scale. There was not much discussion about health content, only mentioning that the information should be from a reliable source.
AppCheck	AppCheck is a German-based evaluation tool for health-related apps. It includes a self-assessment that the app manufacturer fills out and a review of the app by DiaDigital (part of AppCheck). The self-assessment has sections focused on product detail, medical aspects, interaction with users, data management, and transparency of the app. Lastly, there is a technical exam that asks manufacturers about the security and privacy of collected information.	https://appcheck.de/kriterienkatalog	DiaDigital focuses on diabetes apps. Their rating scales cover a lot of diabetes-specific features along with general features that are important to the function and usability of the app.	The majority of the questions are open-ended, and are not on a Likert Scale. Given this, it is difficult to understand what exactly a "good" app is via this tool.

Iprescribeapps	iPrescribeApps is a platform doctors can use to “prescribe” mobile health apps to their patients. The mobile apps are selected by doctors of iPrescribeApps using “evidence-based criteria”. Doctors can search for a chronic condition in iPrescribeApps, and a list of relevant apps will appear. Doctors can pick apps from this list and “prescribe” them to their patients via email. Within this email, a download link for the app is provided along with instructions and videos about how to use the app effectively.	https://www.iprescribeapps.com/	Due to the fact that this tool is powered by physicians, and physicians can “prescribe” these apps, the health content of their recommended apps must be high.	It was not evident what other parameters this tool looks at. For example, there was no scale for security and privacy.
DMD Sante	DMD Santé is a European Union collaboration with a large list of recommendations on health apps and provides information for developers on how their app can become “Certified mHealth Quality”.	https://projet-apiapps.org/en/partners/dmd-sant%C3%A9	DMD Sante goes into detail about safe practices regarding the data privacy and digital security of mobile health apps.	The tool did not cover the accessibility of apps to vulnerable populations.
GPM	GPM is an app that serves curated content to health professionals. They analyzed 800 apps in the French app marketplace. The types of apps included in this selection range from diagnostic tools, to medical dictionaries. This could be a resource for doctors, nurses, and paramedics.	https://www.gpm.fr/actualite.html?id=10093	This tool’s functionalities and medical content are reviewed by health professionals.	These apps do not seem to be for patient use. The apps in their collection are more diagnostic aids.
NHS Framework	The National Health Service (NHS) Framework provides a list of mHealth apps that have been “NHS approved” or are “being tested in the NHS.” This is meant to act as a resource for patients who want to use mobile apps to help manage their conditions.	https://apps.beta.nhs.uk/#	Once on the site, users can click on an app, and will find screenshots from the app itself, along with a description of the functionalities of the app. If there are any issues, this tool provides external links to the app developer’s contact page. Lastly, this tool	The description that they provide is quite brief for some of the apps. There is also no information on the technological requirements of

			continually reviews apps, and are always adding new ones.	each app (i.e. most up to date iOS, how much space will this app need, etc.).
Myhealthapps	Myhealthapps provides patients with a list of apps that have been tested and used by other patients. They receive recommendations from consumers, patients, caregivers, patient groups, charities, and other non-profit organizations. Once myhealthapps has received a recommendation, they assess the transparency of the app. This includes the price, research on the group who recommended the app, and research around the app developer. They also try to get multiple recommendations for one app. As of now, they are in the process of creating a set of user-defined guidelines for mobile health apps, with input from patient groups, health professionals, patients, and experts in this field,	http://myhealthapps.net/methodology	Instead of healthcare professionals recommending apps, patients and other patient advocates are the ones recommending apps. This is a major strength of this tool because the app selection process involves the end-users.	While this toolkit has a great way of selecting apps, the evaluation of these apps is still a little unclear. The specific criteria that will be focused on was not identified yet.
Mental health app framework	The Mental Health App Framework is a guide for health professionals to use when assessing mental health apps. The criteria they focus on includes privacy and security, evidence of user benefit, ease of use, and interoperability.	https://www.psychiatry.org/psychiatrists/practice/mental-health-apps/app-evaluation-model	This framework goes over four main areas that would most definitely help a health professional decide if a mental health app would be beneficial to a patient. Not only does it go over the medical content and ease of use the app, it also discusses the privacy of these apps.	In step 3, “Evidence,” there could be more questions added to help guide the user in deciding whether the information in the app is clinically relevant and accurate.

mHIMMS	The mHIMMS provides general guidelines healthcare providers can use when testing the usability of mobile health apps. The first step is for providers to clearly determine how an app will benefit their practice, and how that app relates to usability. The second step is to refer to resources that may evaluate the app better than the star reviews in the app marketplace (i.e. iMedicalApps.com). Lastly, providers should test several clinical scenarios in these apps to assess its usability. These guidelines recommend that providers should contact the app developers with their results and work with them to improve the usability of their products.	Arellano P, Bochniski J. Selecting a Mobile App: Evaluating the Usability of Medical Applications | HIMSS.; 2012. https://www.himss.org/selecting-mobile-app-evaluating-usability-medical-applications. Accessed July 1, 2018.	This is a very in-depth set of guidelines that would be very useful by healthcare providers. Not only does this guideline cover medical content, it also focuses on the usability of the app, which is important in the ease of use in the app.	These set of guidelines did not cover privacy and security.
Enlight	Created a tool to evaluate eHealth interventions from the lens of quality constructs associated with intervention outcome, such as persuasive design, behavior change, or therapeutic alliance.	https://www.ncbi.nlm.nih.gov/pubmed/28325712	An extremely in-depth tool that covers a lot of topics and areas that relate to the functionality, design, and privacy of mobile health apps. For every section, the scale they use (1 to 5), has descriptions for each level.	Some portions of the tool would potentially need further explanation or even training for raters.
PsyberGuide	PsyberGuide is a mental health app guide that focuses on three core principles: credibility, user experience, and transparency. Their website has a catalog of apps where they provide scores for each of the three constructs, along with other information such as which operating system the app can be used on (Apple vs. Android), and the purpose for each app.	https://psyberguide.org/apps/	The catalog of recommended apps they have is easy to navigate through. Users can click on the score under each construct, and they are brought to a detailed rating where they can see the area that app doesn't perform well on. Along with all this information, apps that are related to one users are	It seems that the raters of each app are trained by PsyberGuide, so it is unclear how this tool might be used in the future.

			looking are presented on the same page.	
ABACUS (App Behavior Change Scale)	ABACUS aims to measure the potential of apps in changing behavior. The tool consists of several constructs that ask about the functionalities of the app that could change behavior.	McKay FH, Slykerman S, Dunn M. The App Behavior Change Scale: Creation of a Scale to Assess the Potential of Apps to Promote Behavior Change. JMIR mHealth and uHealth. 2019;7(1):e11130.	Unlike other app rating scales, ABACUS main focus is on the app's potential ability to change the behavior of the user. With that said, the questions they ask are function-specific, and content-specific. This is an area that other app rating scales do not go into with too much detail.	Each question is worded in a way that only allows for binary responses (Yes or No). For questions that may be more subjective, those responses could use a Likert Scale.
THESIS	THESIS focuses on six main constructs: transparency, health content, technical content, security/privacy, usability, and subjective rating. Under each of these constructs, there were several subdomains. Each of these were rated from 1 (Very Poor Quality) to 5 (Outstanding Quality).		THESIS covers a broad range of topics and subtopics that provide a comprehensive understanding of the app's functionality, design, accessibility, and security. The tool asks about the ease of use for populations with low literacy, and the availability of the app in multiple languages.	Although the tool focuses on diverse populations, it does not address populations that do not have a high level of digital skill.

Supplementary Figure 1. Category 1 (prior study) app selection

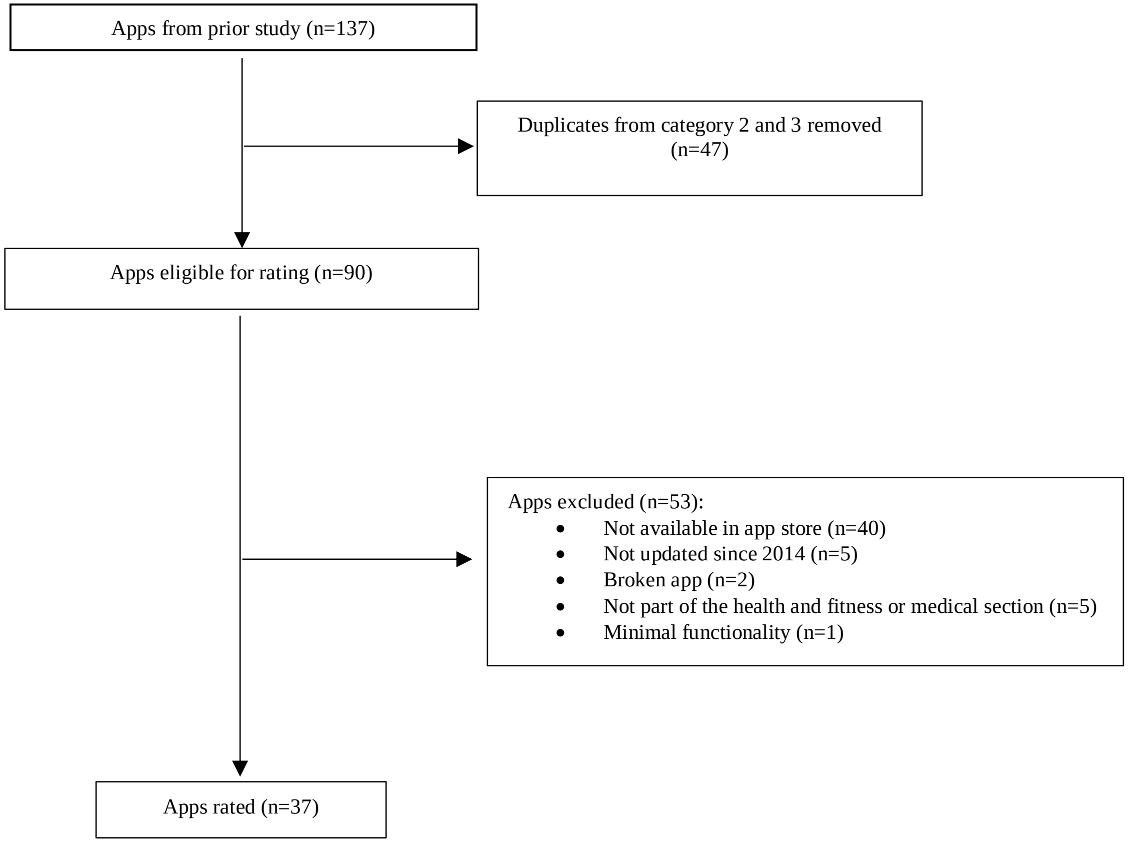

Legend: We rated all the available apps from the previous study

Supplementary Figure 2. Category 2 (4 stars or more) app selection

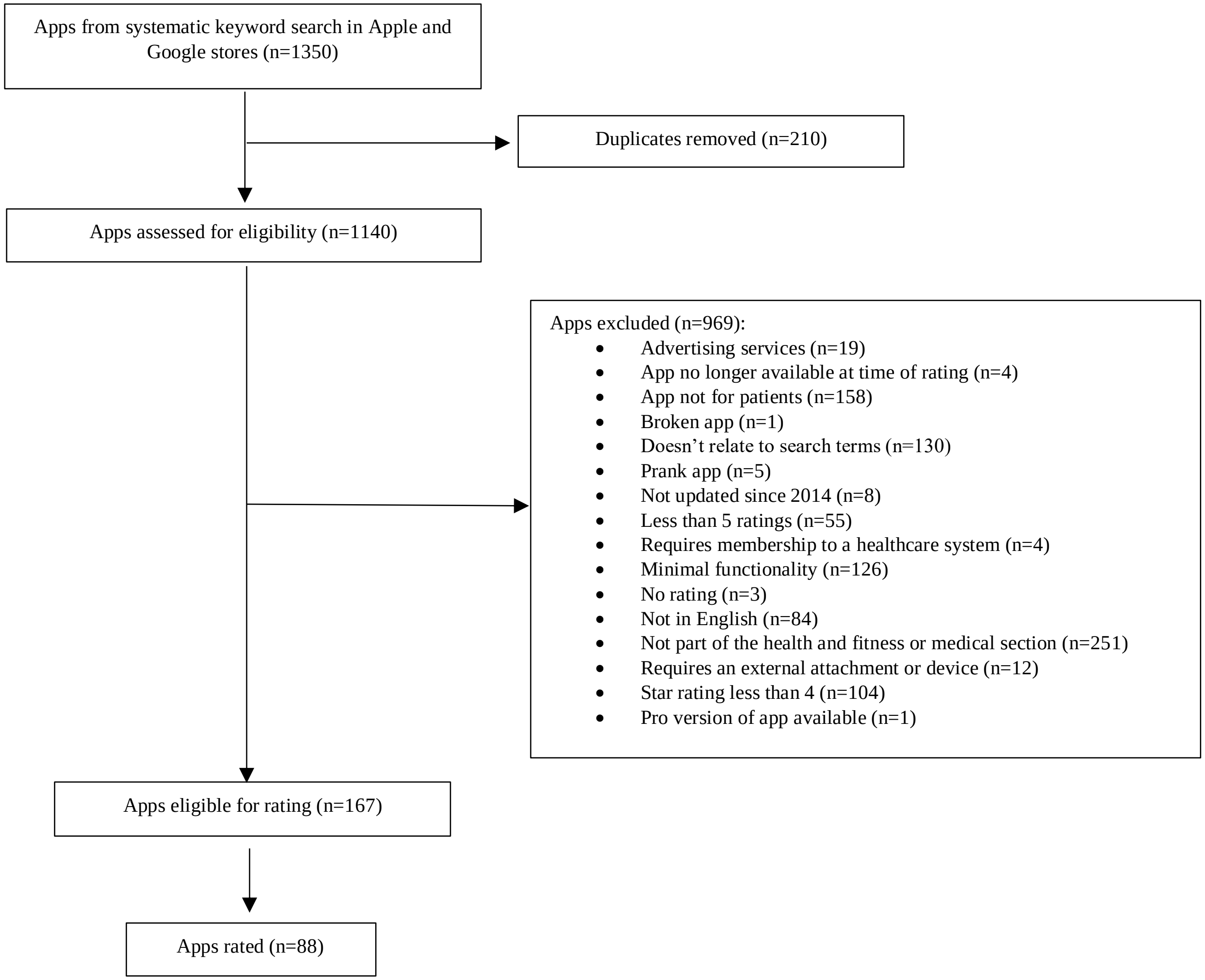

Legend: Not all eligible apps were rated due to resource constraints. We selected the first 4 apps in each disease category.

Supplementary Figure 3. Category 3 (less than 4-star) app selection

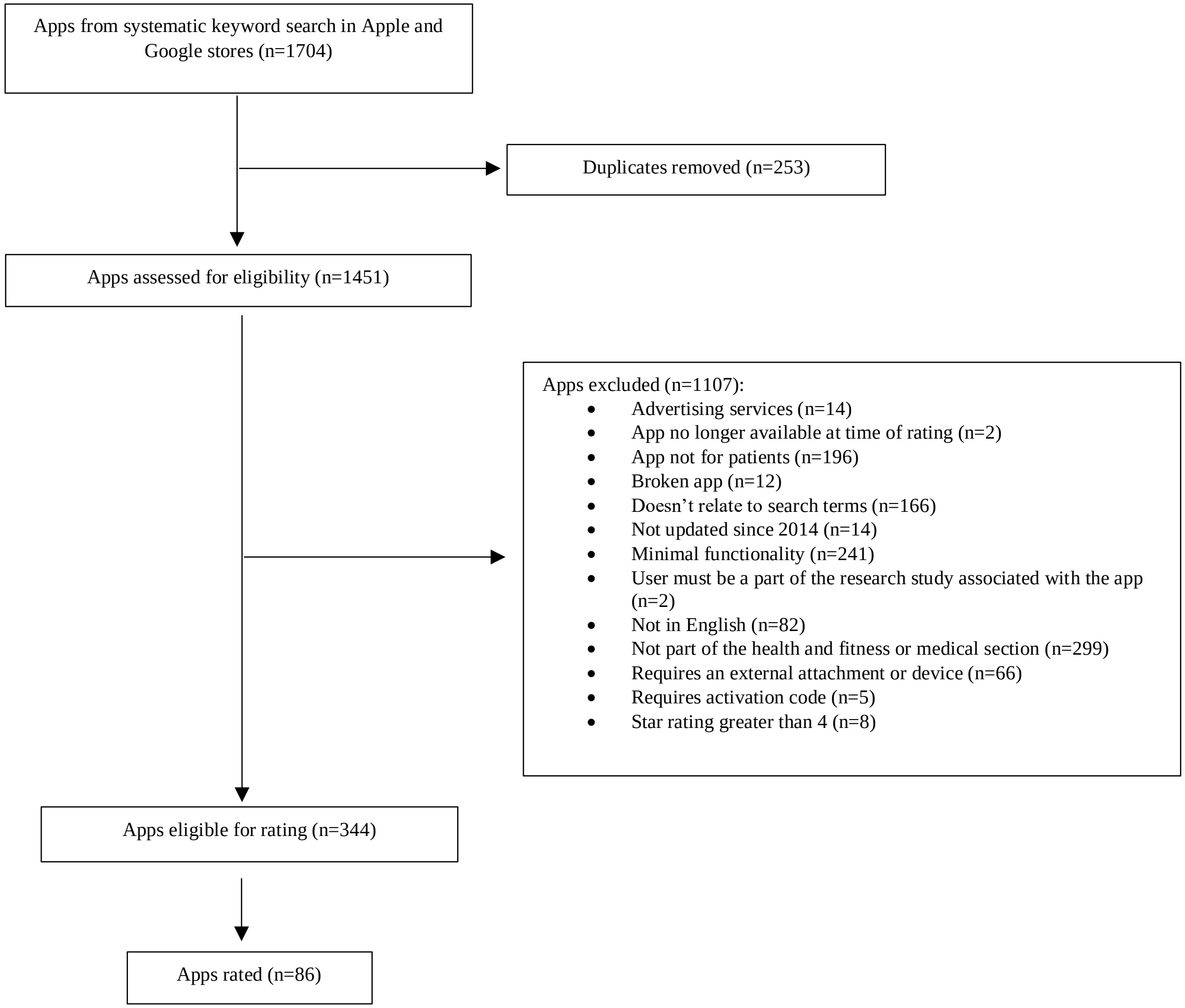

Legend: Not all eligible apps were rated due to resource constraints. We selected the first 4 apps in each disease category.

Supplementary Method 1: Tool iterations during Delphi process

- Transparency
  - ~~Product name reflects purpose~~
    - ~~Does the product name accurately convey the app's purpose?~~
  - Cost of app (purchase price, subscriptions, in-app purchases)
    - Are the prices, subscriptions, and in-app purchases accurately conveyed?
  - Bias (authors' conflicts, funding sources, credibility)
    - Are the authors' conflicts of interest, funding sources, and credibility accurately conveyed?
  - ~~Publisher contact info provided~~
    - ~~How easily can the publishers be contacted?~~
  - Consent
    - What is the quality of the consent process, if any?
  - Accuracy of app store description
    - How accurate is the app store description of the app's purpose?
- Health content
  - Appropriate measurement
    - Does the app appropriately measure what it claims to measure?
  - Appropriate interpretation
    - Does the app appropriately interpret what it claims to interpret?
  - Quality of information
    - How optimal is the quality of information?
  - Quantity of information (scope)
    - How optimal is the quantity of information?
  - ~~Evidence based~~
    - ~~How appropriate is the evidence base presented by the app?~~
  - ~~Update schedule~~
    - ~~How optimal is the app's health content update schedule?~~
  - ~~Open-source vs proprietary algorithms~~
    - ~~Does the app make its algorithms available and open source?~~
  - Potential for harm
    - Is the potential for harm minimized?
  - Literacy level
    - How appropriate is the literacy level for the app's intended audience?
  - Construction of information Presentation of information

        - Is information presented in an optimal manner? For example, is scaffolding used?
- Technical content
    - Software performance/stability
        - Does the app run well with zero interface crashes or bugs?
    - Interoperability
        - Is the app able to exchange information with EHRs and other apps?
    - Bandwidth
        - Does the app require significant bandwidth to run?
    - Application size
        - Does the app require significant storage capacity?
- Security/privacy
    - Clear security statement – bates/levine drop
        - Does the app have a clear security statement?
    - ~~Cryptography~~
        - ~~Does the app follow best-practices in encryption?~~
    - Protection against theft, viruses, etc
        - Does the app have optimal anti-virus and safeguards against breaches?
    - Authentication
        - Is the authentication procedure optimal?
    - Data sharing
        - When sharing information, does the app use best practices?
    - Maintenance
        - Does the app have regular cycles to update and patch its security?
    - Signaling of breaches
        - If a breach occurs, does the app have a method to notify its users?
    - ~~Third-party testing~~
        - ~~Has a third party adequately assessed the app's security?~~
    - Clear privacy statement
    - Anonymisation
        - Does the app appropriately anonymize individuals?
- Usability
    - Installation and setup
        - How would you rate are installation and setup?
    - Engagement: entertainment, interest, interactivity bates/levine override

        - Quality of entertainment, interest, and interactivity?
    - Functionality: ease of use, navigation, gestural design, help/instructions
        - Quality of ease of use, navigation, gestural design, help/instructions?
    - Aesthetics: layout, graphics, visual appeal, image readability
        - Quality of layout, graphics, visual appeal, and image readability?
    - Customization/tailoring
        - Ability to customize and tailor to the specific user's needs?
    - Ease of use for users with disabilities
        - Is the app usable by users with disabilities?
    - Available for offline use
        - How would you rate the app's availability for offline use?
    - Available in multiple languages
        - Is the app available in multiple languages?
    - ~~Technical support available~~
        - ~~How would you rate the ease of obtaining technical support?~~
    - ~~Evaluation by target population~~
        - ~~Was the app evaluated by the target population to optimize usability?~~
    - ~~Satisfaction survey~~
        - ~~Does the app deliver a satisfaction survey to make consistent improvements?~~
- Subjective rating
    - Recommend app
        - Would you recommend this app?
    - ~~Projected use~~
        - ~~How many times a year would you use this app if you were the target audience?~~
    - ~~Would pay for app~~
        - ~~Would you pay for this app?~~
            - ~~Follow-up: How much would you pay for this app?~~
    - Overall star rating
        - What is your overall rating of this app?
    - ~~App store rating~~
        - ~~What is the app store rating for this app?~~

Legend: ~~strikethrough~~ = out because of first round; red = out because of second round; green = newly introduced

Supplementary Method 2: Detailed search criteria

Category 2 and 3 search criteria:

- In the app store, apply the following search terms
    - Hypertension
        - Reflex: blood pressure, high blood pressure
    - Heart failure
        - Reflex: heart disease, congestive heart failure
    - Coronary artery disease
        - Reflex: heart disease, chest pain
    - Cardiac arrhythmias
        - Reflex: abnormal heart rate, bradycardia, tachycardia
    - Hyperlipidemia
        - Reflex: high cholesterol, lipid disorders, high triglycerides
    - Stroke
        - Reflex: CVA (cerebrovascular accident)
    - Arthritis
        - Reflex: joint inflammation, joint pain
    - Asthma
        - bronchial asthma
    - Cancer
        - Reflex: cancer side effects, cancer treatment
    - Chronic kidney disease
        - Reflex: kidney failure, kidney disorders
    - Chronic obstructive pulmonary disease
        - Reflex: emphysema, chronic bronchitis
    - Dementia
        - Reflex: memory loss, Alzheimer's disease
    - Cognitive impairment
        - Reflex: Alzheimer's disease, dementia
    - Depression
        - Reflex: clinical depression, major depressive disorder
    - Diabetes
        - Reflex: high blood sugar
    - Hepatitis
        - Reflex: liver inflammation, hepatitis B, hepatitis C
    - Cirrhosis

- Reflex: liver failure
- Human immunodeficiency virus
  - Reflex: AIDS
- Osteoporosis
  - Reflex: weak bones
- Schizophrenia
  - Reflex: none
- Bipolar disorder
  - Reflex: none
- Substance abuse disorders
  - Reflex: Drug use disorder
  - Alcohol→ reflex: alcoholism
  - Drug abuse→ reflex: heroin, cocaine, crack, speed
  - Smoking
- Pain
  - Reflex: chronic pain, somatization
- If the condition itself does not easily identify health apps, the following suffixes will be addended
  - [insert name of chronic disease] manager
  - [insert name of chronic disease] symptoms
  - [insert name of chronic disease] symptoms tracker
  - [insert name of chronic disease] self-management

Supplementary Method 3: Norming Tool

Mobile App Rating # 1

Medical Mobile App: iTriage

Software/Device: iOS/Apple IPhone

Time Taken: 40 minutes (with note-taking)

- Transparency: **4**

**Sub-Category**	**Score**	**Why not -1**	**Why not +1**
Cost of App	5	Downloading the app is free, and there are no in-app purchases.	N/A
Consent	3	Even though there is only a consent process for sharing health information, that is the most sensitive feature in the app.	The app only asks users for their consent for only one of the features: sharing information with users' health providers. There was no consent process for location sharing.
Accuracy of App-Store Description	4	The app was missing 2 of the features it claimed to have, but that did not take away from the main purpose of the app and its usability	The app description states the app will provide live ER wait times, and user can check in at select facilities<br>▪ These features were not available in the app

- Health Content: **5**

**Sub-Category**	**Score**	**Why not -1**	**Why not +1**
Appropriate Measurement	5	The app produces possible causes of symptoms entered by the patient. Relevant questions are asked about the patient's condition. The first couple questions are very general, and	N/A

		as the patient provides more details about their condition, the more in depth and the questions become.	
Appropriate Interpretation of Data	5	From the patient responses, the app produces a set of possible causes. These causes are completely related to what the user entered.	N/A
Quality of Information	4	All the information provided (description of condition, symptoms, tests, and treatments) has been reviewed by Harvard Medical School (indicated at the bottom of each post). This shows that the content has been reviewed by a reliable outside source.	The images however were a little lackluster in that some of them were very generic and weren't labeled (especially for diagrams of the body)<br>▪ i.e. for "Anal Cancer", the figure showed 4 diagrams of the human reproductive system, but there were no labels.<br>▪ For a patient and/or their caregiver, they might want labeled pictures so they understand the condition better
**Sub-Category**	**Score**	**Why not -1**	**Why not +1**
Potential for Harm	4	This app isn't completely harmless, because the app has the possibility to interpret symptoms incorrectly, which could be dangerous.<br>▪ We did not run into this per say, but erring on the side of caution	Overall the potential for harm is low<br>▪ The app provides a lot of contacts and services for suicide, domestic abuse, etc.<br>▪ Conditions that demand immediate medical

			attention are appropriately labelled<br>▪ When severe symptoms (i.e. shortness of breath) are inputted, pop-up comes up with emergency numbers to call
Literacy Level	4	-The app is mostly readable by general audiences<br>-When symptoms are inputted, correct medical terminology isn't required<br>▪ For example, a user can input "throat hurts" and app can interpret that by presenting symptoms and treatments	In some of the condition descriptions, the app refers to other diseases that users may not be familiar with; don't always provide external links for more information
Presentation of Information	5	-All the information is very well presented, with a lot of icons<br>- To input symptoms, they present a human body (which users can change the gender and rotate) and users can click on the part of the body in pain	N/A

- Technical Content: **4**

**Sub-Category**	**Score**	**Why not -1**	**Why not +1**
Software Performance/Stability	4	Other than the one bug with the medical glossary, the rest of the app ran very efficiently	When the "Medical Glossary" tab is clicked, there is a blank screen.
**Sub-Category**	**Score**	**Why not -1**	**Why not +1**
Interoperability	5	**-Note: this rating may change if personal EHRs were added**	N/A

		- One of the features takes users straight to Google Maps when they want directions to the nearest healthcare provider/ER/Urgent Care, etc. and for the "News" capability, the app takes the user to the website of the news article	
Bandwidth	3	-Some news articles have large images in them<br>-One of the features for symptoms is to click on the part of the body in<br>-Has location services to give users directions to facilities	There are only a few features in the app that require using a lot of data.
Application Size	3	The app size is not between 30 and 40 MB	The app size is not between 10 and 20 MB. The app size is between 20 to 30 MB.

- Security and Privacy: **2**

**Sub-Category**	**Score**	**Why not -1**	**Why not +1**
Protection against Theft, Viruses, etc.	1	N/A	-Didn't find any information regarding the protection against theft and viruses in their privacy and security policies.
Authentication	4	The authentication process they do have in place is secure in that it uses Touch ID and/or a password.	-Only asks for authentication once when the user opens that app
**Sub-Category**	**Score**	**Why not -1**	**Why not +1**
Data Sharing	3	The app claims that all the information shared is only going to be used for the intended purpose; groups they are sharing information with are the users'	-Because the app is in contact with physicians, insurance companies, and other third parties, they do share patient info

		PCPs or other physicians who are trying to help them	▪ They share it for quality purposes, contests, etc.<br>▪ Don't ask for consent directly for use of this information. They state that users' information will only be used for its intended purposes.
Maintenance	3	-The developers seemed to fix the major issues last year and added content, the updates this year seem to be only "minor enhancements".<br>-The last update was not more than 5 months ago	-Last update was 3 months ago, before that was 9 months, and then a year ago→ with in that year, they had updated 8 times<br>-this schedule is not regular right now→ they seemed to fix the major issues last year and added content, the updates this year seem to be "minor enhancements"
Signaling of Breaches	1	N/A	There is no mention of notifying users of breaches in the privacy and security statements
Anonymization	2	They do have a de-identification process<br>• But it is only for some of the location information that they collect<br>• Everything else they collect is not anonymized	-The app collects both Protected Health Information and Additional Health Information→ it is disclosed to multiple parties (3rd parties, healthcare professionals for appointments, their affiliates, etc.)<br>• Due to the nature of the app, users can schedule appointments and they can be in contact with their insurer→ therefore, this

			information is not anonymized

- Usability: **5**

**Sub-Category**	**Score**	**Why not -1**	**Why not +1**
Installation and Set-Up	5	-Very straightforward to download from the app store<br>-Registration is just an email and password	N/A
Functionality	5	-Everything in the app is very clear; the abundance of icons helps a lot<br>- All tabs and sections are clearly marked with their purpose, which makes it easy to navigate	N/A
Aesthetics	4	-Other than the images, the rest of the app is laid out in an organized manner that is visually appealing	-When someone clicks on "images" of a condition, it is difficult to see some of the details, and they also aren't labelled
Customization/Tailoring	5	- The patient can save their insurance info, input their appointments, and list their healthcare providers<br>-Also, patients can save their conditions, procedures, medications, allergies and their searches in the app	N/A
Ease of Use for Users with Low Literacy and Numeracy	3	Patients do not have to use proper medical terminology when they input their symptoms	-A lot of the information is easily understandable for patients and caregivers who have a considerable degree of literacy

			-But the app refers to other conditions while describing a specific condition, without defining the other condition<br>▪ Not everyone knows medical terminology<br>-For someone with low literacy and numeracy, this app would be a little confusing, and at some parts hard to understand
Available in Multiple Languages	5	The app is available in 18 languages.	N/A

- Subjective Rating: **5**

**Sub-Category**	**Score**	**Why not -1**	**Why not +1**
Recommend App	5	-Would recommend this app for anyone who wants to use it see if their symptoms may be something more or a caregiver whose loved one has a condition and they want to know more or get into contact with healthcare providers to treat it<br>-Also a nice reference guide	N/A
Overall Star Rating	4	-But other than those things, the app is very detailed and the information has been backed up by a reliable source<br>-Very easy to use	-Even though the developers heavily marketed the ability of the app to provide real-time ER wait time updates, this feature did not work while using the app<br>-The images they show for conditions are a little vague and the body diagrams aren't labelled<br>• This makes it difficult for patients and

			caregiver if they want to visualize the condition

Mobile App Rating # 2

Medical Mobile App: CareZone

Software/Device: iOS/iPad

Time: 47 min (with note-taking)

- Transparency: **5**

**Sub-Category**	**Score**	**Why not -1**	**Why not +1**
Cost of App	5	Downloading the app is free and there were no in-app purchases or subscriptions	N/A
Consent	5	Every time app asked to scan users' medications or add appointments to their calendar, consent from the user was always required.	N/A
Accuracy of App-Store Description	4	The app description was very accurate. All the features it mentioned were showcased in the app.	N/A

- Health Content: **3**

**Sub-Category**	**Score**	**Why not -1**	**Why not +1**
Appropriate Measurement	4	-To keep patients organized, the app has calendar that sorts out doctor appointments.<br>-The app helps manage medications without manually typing this	N/A

		information in (user scans it into the app)<br>-In helping patients manage their conditions, the app lets users input their blood pressure readings and blood glucose readings.	
Appropriate Interpretation of Data	5	-The rater happened to be taking an antibiotic when rating this app, so they tried to scan their prescription bottle<br>• The app recorded all the info from the prescription label very accurately; didn't miss the major details<br>-When the user inputted their blood pressure or blood glucose readings, the app converted those values into a graph, so patients can monitor their conditions over time.	N/A
Quality of Information	2	-The graph the app produces based on patient entered values is helpful for patients to see trends over time	-While this app is mainly for the user to input their personal info, and keep track of their conditions, it does offer information on insurance and Medicare plans<br>• But once the user click on those, the app says, "might be able to" lower users' plans→ have to call and insurance information<br>• Never out right gives what types of changes the user might see→ immediately directed to calling someone
**Sub-Category**	**Score**	**Why not -1**	**Why not +1**

Potential for Harm	2	-While it is lacking in that specific area, the rest of the app had no potential for harm	-In the "Trackers" feature, users can track their blood glucose, BP, Body temp and other physiological processes<br>• In the blood glucose level tracker, two entries were inputted (200 mg/dL and 210 mg/dL), which are both super high and potentially dangerous<br>• The app merely converted those values into a graph and did not provide any guidance as to how to lower it or emergency numbers just in case<br>• A very similar situation occurred when blood pressure values were inputted
Literacy Level	5	-Much of the information in the app is inputted by the user, and the directions are very simple to read	N/A
Presentation of Information	5	-The information is presented in a very clear and concise manner<br>-Everything is labelled accordingly, a lot of graphics	N/A

- Technical Content: **3**

**Sub-Category**	**Score**	**Why not -1**	**Why not +1**
Software Performance/Stability	5	-There were no crashes or software bugs while using the app	N/A
Interoperability	5	This app has the capability to connect with other apps, but rater did not test because it required the	N/A

		release of personal insurance information	
Bandwidth	5	N/A	-This app doesn't require significant bandwidth.<br>-There are images in the app and icons with animations, but they don't use a lot of data.
Application Size	1	N/A	The app size is over 40 MB.

- Security and Privacy: **4**

**Sub-Category**	**Score**	**Why not -1**	**Why not +1**
Protection against Theft, Viruses, etc.	5	-The app uses the highest level of encryption of the device in use<br>-App will not share personal information without formal requests	N/A
Authentication	3	There is an authentication process in place, that requires a password.	-It was not clear whether there was a separate authentication process for access to insurance information<br>-The app only requires the user to log in only once. Other apps ask for authentication when the app hasn't been used in multiple times; this app only asks once.<br>-No Touch ID
Data Sharing	4	-App tries their best to make sure that information shared with 3rd parties is only used for the intended purpose	-In their privacy statements, they do state that they share data with 3rd parties, but they don't share the process used to make sure the data is safe
Maintenance	5	The app has very regular updates for bugs and new versions, there were at least 2 updates every month	N/A
**Sub-Category**	**Score**	**Why not -1**	**Why not +1**

Signaling of Breaches	5	-Via its security statement, if a breach occurs, the app will notify users with push notifications or when user opens app	N/A
Anonymization	3	-Via the privacy policy, it is up to the user if they want to enter identifiable information	-However, the purpose of the app is to personalize it to the patient, especially in the case with ordering medications, where patients can re-order prescriptions, and if anonymized, the app won’t function to fullest capabilities

- Usability: **4**

**Sub-Category**	**Score**	**Why not -1**	**Why not +1**
Installation and Set-Up	5	- Very straightforward and easy to download<br>-Once the app is downloaded, simply put in email and create password for an account	N/A
Functionality	5	-App is very concise<br>-The menu on the left side has clearly marked features and there are options for help with navigation	N/A
Aesthetics	5	-Very aesthetically pleasing<br>-All graphics are appropriate and help visualize the feature in use	N/A
Customization/Tailoring	5	-The main purpose of this app is to make it personalized for the user<br>• The user can input the medications they are taking, input reminders for doctor’s appointments, and can share it with their families	N/A
**Sub-Category**	**Score**	**Why not -1**	**Why not +1**

Ease of Use for Users with Low Literacy and Numeracy	4	- A lot of graphics available that help visualize what is being said in words	-If the patient has low literacy, it may be difficult for them to accurately input all their information inside the app→ would be dependent on their caregiver
Available in Multiple Languages	1	-App is only available in English	N/A

- Subjective Rating: **4**

Sub-Category	Score	Why not -1	Why not +1
Recommend App	4	- This app is extremely beneficial to patients who want to get in the habit of monitoring their health and would like reminders to take their medications and of doctors' appointments.	This app has a lot of features and push notifications, so it may cause alert fatigue for the patient.
Overall Star Rating	4	-This app is extremely useful for patients who need reminders and need help managing their conditions.	There are safety issues when abnormally high blood glucose and blood pressure are entered, and the amount of alerts and push notifications can cause alert fatigue.

Mobile App Rating # 3

Medical Mobile App: Blood Pressure Monitor- Family Lite

Software/Device: iOS/iPad

Time: 45 minutes (with note-taking)

- Transparency: **5**

Sub-Category	Score	Why not -1	Why not +1
Cost of App	4	-In the app description, it is clearly marked that there are in-app purchases available for users<br>• Lists the feature and the price for that feature (in	- However, while using are in the app, it forces the user to only buy one of the tiers of pricing.<br>• One tier is $2.99, while the other three are $1.99, but

		total 4 tiers of pricing, all <$3.00)	in the app, it only gives users the option to purchase the tier that costs $2.99
Consent	5	- Throughout the app, the terms and condition and privacy statements, they explicitly say that they will always ask for consent before distributing pt information	N/A
Accuracy of App-Store Description	5	-A complete 5 because the app-store description tells users of about all the features, including those that come with the in-app purchases. These features are labeled with "upgrade needed" and the rest are unlabeled and come with the lite version	N/A

- Health Content: **4**

**Sub-Category**	**Score**	**Why not -1**	**Why not +1**
Appropriate Measurement	5	The app records patients' blood pressures, weight, and other things, very effectively.	N/A
Appropriate Interpretation of Data	5	- When a blood pressure reading and a weight is entered by user, it converts that data into a graph and chart accurately; also, puts it into a history table	N/A
Quality of Information	4	-The graph they provide for users is a good visualization of blood pressure trends	-Not a full 5 because even though there is a graph that shows the user's BP over a period of time, there is no information on the correlation (to get that feature, user has to pay)

Potential for Harm	3	-Overall, the app has low potential for harm. The main feature of the app is providing users with a graph that shows their blood pressure trends over time. The app does not function to provide advice or information on high blood pressure.	-When extremely high BPs are inputted, the app only highlights it in red. There is no real follow-up to that information (i.e. having an alert pop up and tell user that this is very dangerous, go to ER, etc.)
Literacy Level	5	-All the information is user entered, and the fields are appropriately labeled.	N/A
Presentation of Information	3	- All the other features of the app ("stats", "history", and "profile") are presented clearly; main issues were only with the graph	- Users cannot zoom into the graphs or expand the size of the graphs. This is in part due to the large side bar that isn't collapsible and the ads at the bottom.<br>• This may make it difficult for users to clearly see the details of the graph.

- Technical Content: **3**

**Sub-Category**	**Score**	**Why not -1**	**Why not +1**
Software Performance/Stability	5	While using the app, there were no crashes or bugs encountered.	N/A
Interoperability	1	N/A	The app has a "Data Sync" feature that allows for automatic data synchronization. It requires the user to set up an account (email and password). An account was set up, but when user attempts to use this feature, there are no "buttons" to push to start running this feature. In addition, there is no section on this in the FAQ section.

Bandwidth	5	N/A	-Doesn't require significant bandwidth at all<br>-The only graphics in the app are the graphs, which do not require any data usage
Application Size	1	N/A	-The app size is more than 40 MB

- Security and Privacy: **2**

**Sub-Category**	**Score**	**Why not -1**	**Why not +1**
Protection against Theft, Viruses, etc.	1	N/A	-There were no mentions of any protection against these things in their privacy and security statements. Those statements mainly talked about how the app will never use personal info without user consent first
Authentication	1	N/A	-To use the BP monitor, there is no need to sign in→ once the app is opened, no password or username is asked and it takes the user straight to the charts<br>-For data sync, a username and password must be created, but after signing in once, the app never asks again
Data Sharing	4	-App does ask for consent to share user information for most cases with 3rd parties	-But at the same time, they cannot guarantee 100% that 3rd parties won't use that information for other purposes
Maintenance	2	- Did have a recent update and they do have updates, just not on a regular basis	-Today (10.26.17), the app was updated<br>-But the update schedule is inconsistent and the gaps between updates is quite long<br>• i.e. Before today, the last update was 1 year ago and

			before that, it was 3 years ago
Signaling of Breaches	1	N/A	- No mention in the security statement or privacy statement of notifying users of breaches
Anonymization	4	- App collects "non-identifying" info such as IP addresses, query info, etc.	- However, if the user visits a 3rd party website via advertisements on their app, they can't confirm that the information will be anonymized

- Usability: **3**

**Sub-Category**	**Score**	**Why not -1**	**Why not +1**
Installation and Set-Up	5	-Very straightforward download from the app store, was able to use app right away	-N/A
Functionality	2	-Other than those things mentioned, the app overall has good functionality<br>-FAQ feature has a lot of helpful answers inside	-When the user wants to start putting in new information, they start at the date. To continue with the rest of the form, a pop-up comes up every time notifying users to fill the other fields. This disrupts the fluidity of using the app and causes alert fatigue<br>-Going between pages such as input, charts, stats, etc. the user must click on a menu on the bottom of the screen. This is located right above a banner of ads, so it is very easy to click on them and exit the app and onto the 3rd party website
Aesthetics	3	-The factors mentioned in the next column to a certain degree take away from the usability of the app, but not to the point where app is very hard to use	-Other than the charts, there are no other graphics in the app<br>-The "Menu", located the left side is way too large and in turn makes the chart smaller

			- Also, b/c it is a free version, there are advertisement banners that also make the charts and other features of the app smaller
**Sub-Category**	**Score**	**Why not -1**	**Why not +1**
Customization/Tailoring	5	-The purpose of the app is to make it so that the user can put in their BP readings and other health related things<br>• Users can do that and add info to each tag in the input menu	N/A
Ease of Use for Users with Low Literacy and Numeracy	3	-To input their personal information, the labels aren't worded difficultly<br>-The answers to FAQs are also worded very simply	-For users with low literacy and numeracy, they may not know what "systolic" and "diastolic" mean and which one is reported on "top" and on the "bottom" on their BP readers<br>• Although one would assume a user downloading this app would know these things, it is better err on the side of caution<br>• If the cg wasn't there to help the user input this information, the user might confuse the systolic and diastolic values
Available in Multiple Languages	2	App is available in more than 4 languages	-The app is available less than 8 languages

- Subjective Rating: **3**

**Sub-Category**	**Score**	**Why not -1**	**Why not +1**

Recommend App	4	-The app is a good, straightforward way for someone who just wants to keep track of their BP, pulse and weight every day→ the graph they provide is very helpful	- However, if the user wants to get more insight on what they are putting in, they should be willing to pay
Overall Star Rating	3	- Overall a simple app to record BP, weight and pulse<br>-Does a simple thing well	-The overall look of the app takes away from one of its main goals, which is to generate graphs for their users to monitor symptoms (menu is too large, graph can't zoom into graph, etc.)<br>-The lack of authentication process is slightly worrisome, especially with data sharing→ (although it didn't work when rater opened the feature) the app doesn't ask users to log in after the first time, so someone could potentially access information in this app and other apps connected to the data sharing feature

Mobile App Rating # 4

Medical Mobile App: Symptom Checker-Possible Diagnosis

Software/Device: iOS/iPhone

Time: 36 minutes (with note-taking)

- Transparency: **4**

Sub-Category	Score	Why not -1	Why not +1
Cost of App	5	Downloading the app was free and there were no in-app purchases	N/A
Consent	1	N/A	There were no consent processes while using this app
Accuracy of App-Store Description	5	The app store describes this app as solely providing possible symptoms and is not a substitute for real medical attention; the app reflects that completely	N/A

- Health Content: **5**

Sub-Category	Score	Why not -1	Why not +1
Appropriate Measurement	5	The app provides a list of possible symptoms based on what the user inputs; the app comes up with possible diagnoses that reflect what was inputted.	N/A
**Sub-Category**	**Score**	**Why not -1**	**Why not +1**

Appropriate Interpretation of Data	5	The diagnoses they provide are a wide range and they provide a lot of different diagnoses based on the user's symptoms	N/A
Quality of Information	5	-The information they provide is written by health professionals (MDs) and they are trained to write the articles in an approachable language<br>-The images they provide are also very informative because of the labels, and are concise.	N/A
Potential for Harm	4	- For the most part, the diagnoses the app outputs do reflect the symptoms the user put in<br>-The app is more geared towards education→ they have a huge disclaimer where the app says users shouldn't use this app in place of true medical help	There is always a chance that the app can misdiagnosis the user. This is illustrated in the app when it produces a list of possible diagnoses. The app doesn't list the diagnoses in the order from most likely diagnosis to least likely diagnosis. A user you could easily misdiagnosis themselves or use information that doesn't directly pertain to their symptoms.
Literacy Level	5	Because the writers of this content are trained professionally to write in an approachable (per their "About Us" section), the language	N/A

		used is easy to read and understand by many audiences.	
**Sub-Category**	**Score**	**Why not -1**	**Why not +1**
Presentation of Information	4	-In the articles about the diagnoses, there are different colored headings for the section along with images that supplement the text	-Along with the images, there are ads that are above or below them, so it is slightly distracting<br>- Also when it presents the list of possible diagnoses, it just lists the names, no brief description<br>▪ For someone who has no idea about any of these terms, they might spend a lot of time clicking through all of them trying to find the diagnosis that fits them

- Technical Content: **2**

**Sub-Category**	**Score**	**Why not -1**	**Why not +1**
Software Performance/Stability	1	N/A	-Several instances where the app froze as symptoms were being put in
Interoperability	1	N/A	The app does not exchange information with EHRs or mobile apps
Bandwidth	1	N/A	-B/c the app has a lot of ads (while putting in symptoms and reading the actual articles), which takes a lot of data. These ads range from just images to videos<br>-Also, there are images and videos that go along with the articles
Application Size	4	Based on our criteria, the application size is not a 3 because it is less than 20 MB.	Based on our criteria, the application size is not a 5, because it is between 10 and 20 MB (12.5 MB).

- Security and Privacy: **2**

Sub-Category	Score	Why not -1	Why not +1
Protection against Theft, Viruses, etc.	1	N/A	The privacy statement did not mention anything about protection against theft and viruses
Authentication	1	N/A	No authentication processes
Data Sharing	5	Per the privacy and security statements, the app does not exchange information with EHRs or other mobile apps	N/A
Maintenance	1	N/A	-Specific information on this could not be located, but it is running on Version 1.5.4, so still on their first version<br>-The app store didn't provide updates on when the last update was for the app
Signaling of Breaches	1	N/A	No indication of how the app would respond to security breaches
Anonymization	5	-Doesn't collect personal information, but users can opt to share their email addresses and zip codes→ this info helps the app improve user experience<br>• If user opts to input this information in, the app's privacy statement says that it will be kept in their secure database and "the data is kept strictly confidential"	N/A

- Usability: **3**

Sub-Category	Score	Why not -1	Why not +1

Installation and Set-Up	5	Installation was very straightforward off the App Store	N/A
Functionality	2	-While there are a lot of ads and interruptions, the app is still usable and users can still access the main features of the app	Because of the number of ads, it makes the app hard to use<br>• After every page, users should expect to go straight to the next page, but a 5-sec ad pops up→ disrupts the fluidity of the app<br>-No FAQ sections<br>-While scrolling through the articles, the ads between and within paragraphs distracts the reader and makes it hard to find their place
Aesthetics	3	The images they provide are labelled and easy to see and the other graphics are of decent quality<br>-The symptoms the app asks the user are laid out nicely in multiple choice form	-The graphic of the body they use for users to click on is a little small<br>• The bottom of it is the disclaimer note by the app developer, so it makes it smaller<br>-Everything is in list form and large paragraphs, which makes it a little overwhelming to look at
Customization/Tailoring	4	The user themselves put their own symptoms in and the result are diagnoses that should match what the user inputted	-There is a portion of the article describing treatments that provides treatments (i.e. list of drugs). Patients cannot put in allergies to different meds
Ease of Use for Users with Low Literacy and Numeracy	5	The articles don't use complex wording and don't overuse medical terms, so it is readable by everyone	N/A

Sub-Category	Score	Why not -1	Why not +1
Available in Multiple Languages	1	N/A	Only available in one language (English)

- Subjective Rating: **3**

Sub-Category	Score	Why not -1	Why not +1
Recommend App	2	The app does give users good, concise information that is readable by a large audience	-The number of ads take a lot away from the main purpose of the app, to the point where it becomes annoying to use. There are other apps that do the same thing without the interruptions<br>-The aesthetics aren't the best and again there are apps that do the same and their images and graphics are very clear
Overall Star Rating	3	For what the app is, it does accomplish its goal<br>▪ The app does give users good, concise information that is readable by a large audience and provides appropriate images that supplement those descriptions	-There are too many ads and interruptions in this app and it crashes frequently<br>-There are no frequent updates on the app itself→ may be what is causing those crashes

Mobile App Rating # 5

Medical Mobile App: medic-app

Software/Device: iOS/iPad

Time: 27 minutes (with note-taking)

- Transparency: **4**

**Sub-Category**	**Score**	**Why not -1**	**Why not +1**
Cost of App	5	There are no in-app purchases, and the app is free to download.	N/A
Consent	1	N/A	There are no consent processes used in this app
Accuracy of App-Store Description	5	App store description says that user can review their condition and learn about them, and from using the app this is accurate	N/A

- Health Content: **2**

**Sub-Category**	**Score**	**Why not -1**	**Why not +1**
Appropriate Measurement	3	There are few select questions that a bit more specific.	Most of the questions asked about the users' symptoms are very broad and many possible diagnoses can be made from the answers to these questions.
Appropriate Interpretation of Data	1	N/A	-Once the user enters their symptoms, the app doesn't interpret them very accurately.<br>-Some of the diagnoses the app gives are very severe<br>▪ i.e. clicked on the right upper back on the body, then said had pain, then inputted had fatigue and then the app responded with a diagnosis of smallpox
**Sub-Category**	**Score**	**Why not -1**	**Why not +1**

Quality of Information	2	-The app does say the information on the diseases are reviewed by a medical source and after reading them, they do seem legitimate	-Per the app's statements, the information about the individual diseases have been reviewed by a medical source<br>• But there is no information on the medical sources they received the information from.<br>- When a user clicks on one of the drugs under the drugs and supplements tab, there are no descriptions what the exactly does.
Potential for Harm	1	N/A	- The potential for harm is very high since most of the diagnoses produced are either very general diagnoses or very extreme<br>-Also, the app doesn't give information the exact functions of the drugs and supplements; they only provide the name. Potentially, users may take them without knowing any of the risks and have no information on whether the drug will help relieve some of their symptoms
Literacy Level	2	When the app asks the user about their symptoms, the app uses very simple language→ easy to understand	The descriptions of the diseases use some complex medical terms that everyday users probably don't know; they app also doesn't provide links for more information on those
Presentation of Information	3	The presentation of the information is clear and concise→ nothing is hard to read or see	-No scaffolding used, the descriptions are in paragraph form, no images to break any of that information up.

			-There is a lot of "white space" in the app, so the developers could have used that space to provide images

➢ Technical Content: **3**

**Sub-Category**	**Score**	**Why not -1**	**Why not +1**
Software Performance/Stability	1	N/A	The app quits by itself several times while being used→ user had to re-open the app each time
Interoperability	1	N/A	This app does not exchange information with EHRs or other mobile apps
Bandwidth	5	Doesn't require much bandwidth→ no images, animations, or videos that use data; the only graphic used for users to click which part of the body is in pain	N/A
Application Size	5	Very small app size (9.8 MB), compared to the other apps being reviewed	N/A

➢ Security and Privacy: **1**

**Sub-Category**	**Score**	**Why not -1**	**Why not +1**
Protection against Theft, Viruses, etc.	1	N/A	App doesn't have a security statement, and this item was not mentioned in their terms and privacy
Authentication	1	N/A	No authentication processes
Data Sharing	1	N/A	App doesn't share data with any third-party sources or other apps

Maintenance	1	N/A	No frequent updates for the app, the updates that were recorded were multiple years apart
**Sub-Category**	**Score**	**Why not -1**	**Why not +1**
Signaling of Breaches	1	N/A	The app doesn't provide any information on if it will notify user of breaches
Anonymization	3	At the same time, the app doesn't ask for any identifiable information from user<br>▪ User doesn't need to enter a name, email address or other information like that to use the app	Because the app doesn't provide users with security statements, there are no explicit statements outlining their anonymization or if there even is one

- Usability: **2**

**Sub-Category**	**Score**	**Why not -1**	**Why not +1**
Installation and Set-Up	5	Downloading and setting up the app is very straightforward	N/A
Functionality	2	The individual menus are easy to read and there is no confusion what each menu item is referring to	-User cannot go to previous answers/pages if they make a mistake answering questions about their symptoms→ they must start the entire process over<br>-There is no fluidity in navigating through the menus; constantly going back and forth<br>-There is no FAQ or help section
Aesthetics	1	N/A	-Not visually appealing<br>-The layout is all separate menus<br>-The graphic of the body they use resembles a sketch and the lines are unsteady (not sharp)<br>-There are no images to supplement the information
**Sub-Category**	**Score**	**Why not -1**	**Why not +1**

Customization/Tailoring	1	N/A	-Users can input symptoms that relate to them, but **only** the ones that the app offers<br>-If user has allergies, there aren't any options to enter those<br>-All the questions about symptoms are multiple choice, there are no free response questions; everything is based on the information programmed into the app
Ease of Use for Users with Low Literacy and Numeracy	2	The symptoms and menu features use simple language, where it is not difficult to understand what they mean	Once user starts reading about the diseases, the app uses a lot of medical terms and refer to a lot of significant values that relate to the disorders (i.e. high BP is 140/90 mmHg). They do not provide further resources to help explain these terms (i.e. external links or labelled images)
Available in Multiple Languages	1	N/A	The app is only available in 3 languages.

- Subjective Rating: **2**

**Sub-Category**	**Score**	**Why not -1**	**Why not +1**
Recommend App	1	N/A	Would not recommend this app. The medical information that is provided for users isn't backed up by any identifiable medical organization or reputable source. In addition, the diseases the app diagnoses users with are not accurate and are extreme cases. Also, the overall aesthetics and functionality left a lot to be desired and affected the usability of the app
**Sub-Category**	**Score**	**Why not -1**	**Why not +1**

Overall Star Rating	2	The app is useful for maybe looking up the details of some diseases. But users should err on the side of caution with this because the app doesn't provide information about which medical they source they used to find this information	The lack of customization, along with the inaccurate diagnoses the app produces are the glaring flaws. In addition, there is a huge lack of information about the app developers themselves and they also do not provide users with security and privacy statements

Mobile App Rating # 6

Medical Mobile App: AskMD

Software/Device: iOS/iPhone

Time: 54 min (with note-taking)

- Transparency: **5**

**Sub-Category**	**Score**	**Why not -1**	**Why not +1**
Cost of App	5	The app was free download, and there were no in-app purchases	N/A
Consent	5	-The app works in conjunction with Apple's Health app, and the app	N/A

		asks if they can share the user's personal info with them<br>-The app also asks user if they can use their location	
Accuracy of App-Store Description	4	-Other than what was mentioned, the app description is very accurate	-When severe symptoms such as "heart attack" were inputted and then added on "severe chest pain", the app should have popped up with a "critical or life-threatening" notification. This feature was not encountered while using the app.

- Health Content: **4**

**Sub-Category**	**Score**	**Why not -1**	**Why not +1**
Appropriate Measurement	5	-After patient inputs their symptoms, the app produces a list of likely and unlikely causes	N/A
Appropriate Interpretation of Data	4	For the most part, the causes that the app comes up with are accurate and have something to do with the specific set of symptoms that the user reports	When more severe cases are inputted, the causes that the app gives are probable. But, if a user reports "heart attack", the app should interpret it as a severe condition and notify the user of nearby ERs (like it said it would in their description)
Quality of Information	5	All the information about the causes/symptoms/treatments are cited appropriately and the app has a "references" section in each of the descriptions of the diseases→ most of their sources are textbooks, FDA's website, and journals	N/A
Potential for Harm	4	The app does provide some critical support (in addition to the example mentioned earlier)	-This app has the potential for harm if it interprets the symptoms

		• For example, the "Mental Health Check-Up", immediately produces a warning to call the suicide hotline when patients input symptoms that could lead to suicide	incorrectly and diagnoses users with the wrong cause<br>-The app doesn't always alert user of critical conditions and symptoms
**Sub-Category**	**Score**	**Why not -1**	**Why not +1**
Literacy Level	3	There are only a couple of instances where medical terminology is used. The rest of the app does not use medical terminology.	-For the description of causes and treatments, the app uses a good amount of medical terminology and refer to other diseases (i.e. cytomegalovirus)<br>-The app does not provide an "info" button for all cases where the user might not know the term(s)
Presentation of Information	5	-Scaffolding is used<br>-Labels for each section within each diagnosis<br>-Everything presented in an organized manner→ for unfamiliar terms (i.e. tremors), the app immediately defines it underneath the question about user's symptoms<br>• This makes it easy for users to have access to that information	N/A

- Technical Content: **4**

**Sub-Category**	**Score**	**Why not -1**	**Why not +1**
Software Performance/Stability	5	While using the app, there were no crashes or software bugs	N/A
Interoperability	5	This app does exchange information with Apple's Health and Maps apps.	N/A

		• Exchanges those types of information with consent and the app does not crash when that information is being exchanged	
**Sub-Category**	**Score**	**Why not -1**	**Why not +1**
Bandwidth	3	The app does not have any images or videos that require a lot of data usage	-Because the app does use Location services which requires some data usage (depending if user is using Wi-Fi or not)<br>-To look up all the different causes, the app does use the internet, which requires the use of cellular service
Application Size	1	N/A	The app's size is greater than 40 MB (the app's size is 41.5 MB).

- Security and Privacy: **4**

Sub-Category	Score	Why not -1	Why not +1
Protection against Theft, Viruses, etc.	5	Their security statements along with other privacy statements explicitly mention how they protect against these threats<br>• Encrypt many of their services using SSL<br>• Review their information collection, storage and processing methods along with physical measures to protect against unauthorized use of their systems<br>• All personal information collected is restricted to use only by their	N/A

		employees and their contractors and agents	
**Sub-Category**	**Score**	**Why not -1**	**Why not +1**
Authentication	5	Every time the user closes app and then re-opens it, app will ask for either a password, pin number, or TouchID before using the app (depends on which of those the user has set up)	N/A
Data Sharing	5	The app will ask for user's consent before sharing their information with other services such as third parties used by the app to measure user experience and app usage	
Maintenance	4	There is a consistent update cycle and the bug they fixed for the last one makes the app run very smoothly	Their update cycle/pattern seems to be every year; could be more frequent to fix bugs
Signaling of Breaches	1	N/A	There wasn't mention of the app's response to signaling breaches
Anonymization	4	In their security and privacy statements, states that they collect anonymous identifiers and will always ask for consent to de-anonymize personal info<br>• The user can also control who has access to their information→ able to set up in their Privacy settings	The app isn't fully able to use unique identifiers on everything<br>• When users are using location-enabled services, the app will collect the user's exact location<br>• App is also able to collect and store some personal

		• The anonymous identifiers are used when the user uses services offered by partners of Sharecare (the app developer)	information locally (meaning on the user's device)

- Usability: **4**

**Sub-Category**	**Score**	**Why not -1**	**Why not +1**
Installation and Set-Up	5	The app was easy to download and setting an account was very straightforward.	N/A
**Sub-Category**	**Score**	**Why not -1**	**Why not +1**
Functionality	5	-The app takes the user step by step through all the menus<br>-Everything is labelled appropriately so user knows which part of the consultation they are on	N/A
Aesthetics	5	-Font size, color scheme, and layout of the app are all tastefully done.<br>-The design of the app does not inhibit or distract the user from using the app to its fullest capabilities	N/A
Customization/Tailoring	4	-Overall, the app customizes features to the user and all the symptoms are self-reported<br>• The symptom questions are a mix of multiple choice and fill-in the blank<br>-The app also produces a page of what was inputted by the user, which the user can bring with them to their doctor→ extremely convenient	-App does not allow the user to save some of the information that it asks repeatedly in the symptoms section<br>• i.e. for a lot of the symptoms, app asks if the user has ever been outside of the US before. The app does not allow users to pre-input that information and save the info; users must keep inputting that repeatedly

Ease of Use for Users with Low Literacy and Numeracy	3	-Overall the language used is approachable and someone with low literacy can navigate through it<br>-The app also allows users to input information for someone else→ i.e. someone with low literacy can have their cg do it for them	-However there is a decent amount of medical terms and medical diseases used to describe symptoms.<br>-Information buttons explaining these aren't always available
Available in Multiple Languages	1	N/A	App is only available in English

- Subjective Rating: **4**

**Sub-Category**	**Score**	**Why not -1**	**Why not +1**
Recommend App	5	The app is extremely easy to use and asks in-depth questions about the user's symptoms. The causes the app produces correlate to what was inputted by the user<br>-Also a great tool to keep track of a list of symptoms when user goes to see the doctor	N/A
Overall Star Rating	4	The app is extremely easy to use, the symptom checker is very detailed (all the questions it asks have direct connection to the main symptom the user inputted), and the diagnoses the app produces are consistent with the user's symptoms. In addition, all the medical information is cited appropriately and the references are given to the users.	Just missing a few features that it mentioned in the app store description and the wording used by the app may not be readable by all audiences.
